# Machine learning study using 2020 SDHS data to determine poverty determinants in Somalia

**DOI:** 10.1038/s41598-024-56466-8

**Published:** 2024-03-12

**Authors:** Abdirizak A. Hassan, Abdisalam Hassan Muse, Christophe Chesneau

**Affiliations:** 1 School of Postgraduate Studies and Research, Amoud University, Amoud Valley, Borama, Awdal 25263 Somalia; 2 Department of Mathematics, LMNO, CNRS-Université de Caen, Campus II, Science 3, 14032 Caen, France

**Keywords:** Machine learning, Somalia, Random forest, Model precision, Classical regression, Sustainability, Demography, Energy science and technology, Mathematics and computing

## Abstract

Extensive research has been conducted on poverty in developing countries using conventional regression analysis, which has limited prediction capability. This study aims to address this gap by applying advanced machine learning (ML) methods to predict poverty in Somalia. Utilizing data from the first-ever 2020 Somalia Demographic and Health Survey (SDHS), a cross-sectional study design is considered. ML methods, including random forest (RF), decision tree (DT), support vector machine (SVM), and logistic regression, are tested and applied using R software version 4.1.2, while conventional methods are analyzed using STATA version 17. Evaluation metrics, such as confusion matrix, accuracy, precision, sensitivity, specificity, recall, F1 score, and area under the receiver operating characteristic (AUROC), are employed to assess the performance of predictive models. The prevalence of poverty in Somalia is notable, with approximately seven out of ten Somalis living in poverty, making it one of the highest rates in the region. Among nomadic pastoralists, agro-pastoralists, and internally displaced persons (IDPs), the poverty average stands at 69%, while urban areas have a lower poverty rate of 60%. The accuracy of prediction ranged between 67.21% and 98.36% for the advanced ML methods, with the RF model demonstrating the best performance. The results reveal geographical region, household size, respondent age group, husband employment status, age of household head, and place of residence as the top six predictors of poverty in Somalia. The findings highlight the potential of ML methods to predict poverty and uncover hidden information that traditional statistical methods cannot detect, with the RF model identified as the best classifier for predicting poverty in Somalia.

## Introduction

### Background of the study

Poverty reduction has become a crucial global mission, particularly for countries facing significant economic challenges^1^. The United Nations (UN) has outlined 17 Sustainable Development Goals (SDGs) for 2015–2030, with the eradication of all forms of poverty being a key objective^1,2^. Despite global efforts, a World Bank report in 2020 revealed that 10% of the world’s population still lived in poverty^3^. In this context, monitoring poverty and identifying its determinants are essential for policymakers and researchers to understand the living conditions of the poor and develop effective poverty reduction strategies^4^.

Sub-Saharan Africa, despite being rich in natural resources, remains grappling with widespread poverty. The region faces numerous challenges, including limited access to quality education, healthcare, and basic infrastructure. High levels of unemployment, income inequality, and food insecurity further exacerbate the poverty situation^5^. Sub-Saharan Africa is also particularly vulnerable to external shocks such as climate change, economic downturns, and political instability, which further hinder poverty reduction efforts. Attempts to alleviate poverty in the region require comprehensive strategies that address the multifaceted nature of the problem and prioritize sustainable development and inclusive growth^6^.

Somalia, situated in the Horn of Africa, faces its own unique set of challenges in combating poverty. The country has experienced decades of political instability, armed conflict, and recurring droughts, which have severely impacted its socio-economic landscape. Poverty rates in Somalia are among the highest in the region, with a significant proportion of the population living below the poverty line. Limited access to basic services, such as healthcare and education, further exacerbates the poverty situation^7^. Additionally, the country’s vulnerability to climate change and ongoing security concerns present additional obstacles to poverty reduction efforts^1^. According to the Voluntary National Review report (2022), poverty rates in Somalia are remarkably high, with nearly seven out of ten Somalis living in poverty, making it the sixth-highest rate in the region. Among nomadic pastoralists, agro-pastoralists, and internally displaced persons (IDPs), poverty stands at an average of 69%. Conversely, urban areas exhibit a comparatively lower poverty rate of 60%. These figures underscore the gravity of the situation. Addressing poverty in Somalia requires a comprehensive approach that addresses the root causes of instability, promotes inclusive governance, and focuses on sustainable development strategies that prioritize the well-being of its citizens.

In the realm of poverty research, classical regression models have been the predominant analytical approach utilized by scholars and practitioners^8^. Linear regression models, in particular, have been commonly employed to explore the relationship between poverty and various socio-economic variables^9^. Logistic regression models have also been widely utilized to understand the determinants of poverty and identify significant predictors^10^. These traditional regression techniques have provided valuable insights into the factors associated with poverty and have contributed to the existing body of knowledge. However, it is important to realize that poverty is a complex phenomenon influenced by a multitude of interconnected factors. As such, there is a growing recognition of the need to explore alternative methodologies that can capture the inherent non-linearities and intricate relationships within poverty dynamics^11^.

Recent studies within the field of poverty research have increasingly turned to machine learning (ML) techniques for poverty prediction and analysis^4^. The ML algorithms, such as random forest (RF), decision tree (DT), support vector machine (SVM), and logistic regression, offer distinct advantages in capturing complex patterns and relationships within large and diverse datasets^12–14^. These techniques have the potential to uncover hidden insights and identify novel determinants of poverty that may have been overlooked by traditional regression models. By harnessing the power of ML methods, researchers are able to develop predictive models that can accurately forecast poverty levels and contribute to more targeted and effective poverty alleviation strategies. The integration of these modern methods into poverty research marks an important step, allowing for a deeper understanding of the nuanced dynamics and causal factors that underlie poverty and opening new avenues for evidence-based policy interventions^15,16^.

Despite the pressing need to understand the key determinants of poverty in Somalia, there has been a significant gap in research focusing on this issue due to the limited availability of comprehensive datasets. However, with the introduction of the first-ever Somali Demographic and Health Survey (SDHS) data in 2020, an opportunity has emerged. Our study aims to fill this gap by utilizing the SDHS data to identify and analyze the key determinants of poverty in Somalia. We employ a multidimensional approach, utilizing both classical regression models and accurate ML algorithms such as RF, DT, SVM, and logistic regression to predict poverty levels. By combining these methodologies, we seek to provide valuable insights into the factors driving poverty in Somalia, facilitating evidence-based policy interventions and targeted poverty reduction strategies.

By conducting this study, we aim to contribute to the existing literature on poverty reduction by providing insights into the specific determinants of poverty in Somalia. The findings will not only enhance our understanding of the factors influencing poverty but also inform policymakers and development practitioners about designing targeted interventions to alleviate poverty in Somalia. Furthermore, we present the potential of the ML techniques in poverty prediction, highlighting their applicability in data-scarce settings like Somalia.

### Determinants of poverty: a global to country-level review

This review aims to emphasize on the multifaceted nature of poverty and its determinants, examining both common global trends and the unique dynamics within Africa, East Africa, and Somalia. By exploring these different levels of analysis, a comprehensive understanding of the factors driving poverty can be developed, allowing for more targeted interventions and policies to alleviate poverty and improve socio-economic conditions.

Provide a brief overview of the global perspective on poverty and its determinants sets the stage for understanding the bigger picture. Kaplinsky^17^ and Bracking^18^ both highlighted the adverse impact of globalization on poverty, with the former emphasizing the role of global economic systems in perpetuating poverty and the latter underscoring the adverse terms of integration into the global economy. Sumner^19^ further naunced the picture by pointing out that poverty is not solely a result of a lack of resources but is also influenced by national inequality, social policy, and economic development. Neutel^20^ provided a more optimistic view, suggesting that globalization can reduce poverty and income inequality, although the relationship is not straightforward. These studies collectively underscore the need for a comprehensive understanding of the various factors that contribute to poverty in the global context^21^.

Focusing in on Africa, we examine the specific factors that contribute to poverty across the continent, considering both common trends and regional variations. Adeyemi^22^ identified population growth, inflation, external debt, lack of safe water, low economic activity, gender discrimination, ethnic and religious conflicts, and HIV/AIDS as key determinants of poverty in Sub-Saharan Africa. Akanbi^23^ emphasized the role of governance and physical infrastructure in poverty reduction, with better governance and infrastructure leading to lower poverty levels. Sackey^24^ underscored the importance of education, household characteristics, economic activity, and access to capital in poverty reduction. Kabuya^6^ highlighted the role of pro-poor policies, weak economic and political institutions, and culture as fundamental causes of poverty in the region.

Narrowing further to East Africa, we examine the unique dynamics and determinants of poverty within this specific region, which may differ from other parts of the continent. Binam^25^ highlighted the importance of access to infrastructure and village resources, while Teka et al.^26^ emphasized the role of economic growth. Addae-korankye^27^ added to this by identifying corruption, poor governance, limited employment opportunities, and poor resource usage as key factors. Anyanwu^28^ further underscored the impact of income inequality, education, mineral rents, inflation, and population size on poverty levels. These studies collectively suggest that a multi-faceted approach, including investment in infrastructure, economic growth, and governance reforms, is needed to address poverty in East African countries.

Finally, we explore the case of Somalia, focusing on the country-level factors that shape poverty and its determinants within its specific socio-economic and political context. Mohamoud^29^ found that household size, education level, access to electricity, and engagement in small family businesses, agriculture, fishing, and hunting significantly influence the likelihood of poverty. Muktar^30^ highlighted the impact of access to irrigation, distance from market centers, farm land size, non-farm activities, educational status, livestock holding, and herd diversification on poverty among agro-pastoral households. Ali^31^ emphasized the role of household head gender, age, marital status, credit acquisition for food, main source of food, and seed shortage in determining food insecurity. Pape^32^ underscored the significant impact of drought on poverty, particularly in rural areas. The combined findings of these research indicate that a combination of socio-economic, environmental, and geographical factors contribute to poverty in Somalia^7,33,34^.

### Motivation

The motivation behind this study comes from the urgent need to address the persistent and widespread issue of poverty in Somalia. Despite various efforts and initiatives, poverty remains a significant challenge, affecting a large proportion of the population and hindering the country’s overall development. The Sustainable Development Goals (SDGs) provide a comprehensive framework to tackle poverty and promote sustainable development globally. However, it is essential to assess the progress made by individual countries, such as Somalia, towards achieving these goals.

On the other hand, ML methods have emerged as powerful tools for predicting and understanding complex phenomena. While they have been successfully applied to poverty analysis in other developing countries, there is a lack of research utilizing these methods specifically in the context of Somalia. By applying ML algorithms, to predict poverty in Somalia, we can leverage their predictive capabilities of these advanced techniques and gain insights into the key factors driving poverty in the country.

### Novelty of the study

To the best of our knowledge, this study represents the first attempt to apply ML methods to predict poverty in Somalia. While previous research has explored this subject in the country using conventional regression analysis, the utilization of advanced ML techniques provides a novel approach to understanding and predicting poverty patterns^29,35^. By employing ML algorithms such as RF, DT, SVM, and logistic regression, we can uncover hidden information and complex relationships between predictors and poverty outcomes that may not be captured by traditional statistical methods. Furthermore, our study extends the analysis to evaluate the country’s progress towards achieving the Sustainable Development Goal related to poverty reduction. By examining the specific predictors of poverty and evaluating the performance of different predictive models, our research contributes to the existing literature on poverty analysis in Somalia. Furthermore, it provides valuable insights for policymakers and stakeholders in their efforts to address poverty and promote sustainable development in the country.

### Outline of the paper

The remaining sections of this paper are structured as follows: In "Materials and methods" Section, we provide a detailed description of the methodology employed in this study. This includes the data collection and preprocessing procedures, feature selection techniques, model development, and evaluation of model performance. The results of our analysis are described in "Discussion" Section. "Conclusion" Section focuses on the discussion of the key findings, their interpretation, and their implications in the context of poverty reduction in Somalia. "Limitations of the study" Section summarizes the implications of our research and proposes avenues for future studies aimed at addressing poverty reduction in Somalia. Section Policy implications discusses the limitations of the study. Finally, Section Policy implications presents the policy implications of the study.

## Materials and methods

### Study design and data collection

This study utilized a population-based cross-sectional study design, and the primary data source was the 2020 SDHS. The SDHS is a nationally representative survey conducted by the Somalia Ministry of Health and the Somalia National Bureau of Statistics in collaboration with international partners. The survey collected comprehensive data on various socio-economic and demographic indicators, including household characteristics, income, education, health, and poverty-related variables.

### A geographical overview of the study area

Somalia, located in the Horn of Africa, encompasses a land area estimated at 637,657 km^2^ and exhibits diverse geographical features. Its terrain mainly consists of plateaus, plains, and highlands, offering a varied landscape. Notably, Somalia boasts the longest coastline in Africa, stretching over 3333 km along the Gulf of Aden to the north and the Indian Ocean to the east and south. Its neighboring countries include Djibouti to the northwest, Ethiopia to the west, and Kenya to the southwest.

Characterized by a tropical hot climate, Somalia experiences minimal seasonal variations, with daily temperatures ranging from 30 to 40 ^∘^C. The country observes distinct seasons, with the rainy periods known as Gu’ and Deyr, and the dry seasons referred to as Haga and Jilal. However, Somalia has faced challenges stemming from changing and unpredictable climate patterns, leading to recurrent floods and droughts that impact various regions within the country^7,32,34^.

### Sample in the study

To enhance the quality of life for the Somali population, an extensive survey was conducted across two phases of the SDHS, covering a total of 100,000 households. The survey specifically aimed to capture the perspectives of nomadic communities as well as individuals residing in urban and rural areas, with a focus on understanding their unique needs and challenges. This study includes comprehensive information from 32,298 households, providing a robust dataset for analysis and informing targeted interventions to improve the overall well-being of Somalis.

### Variables in the study

#### Outcome variable

The outcome variable is the wealth index, denoted as *WI*, which serves as a comprehensive measure of a household’s overall wealth. A detailed explanation of its construction can be found in the supplementary information (available at the Somalia National Bureau of Statistics website). The original index consists of five categories: “poorest,” “poor,” “middle,” “richer,” and “richest.” In our study, we redefined the dependent variable as a binary variable, represented by *Y*. Specifically, the categories “poorest” and “poorer” were assigned a value of 1, indicating relative poverty within the household. On the other hand, the categories “middle,” “richer,” and “richest” were grouped together and assigned a value of 0, indicating relative affluence within the household.

#### Predictor variables

After removing the variables used as components in constructing the wealth index (the dependent variable), we preserve the remaining variables that could potentially have associations with poverty. These variables encompass the administrative region, household size, age group of respondents, employment status of the husband, age of the household head, place of residence, education level of the husband, exposure to mass media, sex of the household head, source of drinking water, maternal education, type of toilet facility, and employment status of the mother.

### Data preprocessing

The raw data obtained from the SDHS underwent preprocessing to ensure its suitability for analysis. This included data cleaning, missing value imputation, and variable transformation if necessary.

#### Data cleaning

**Figure 1:**
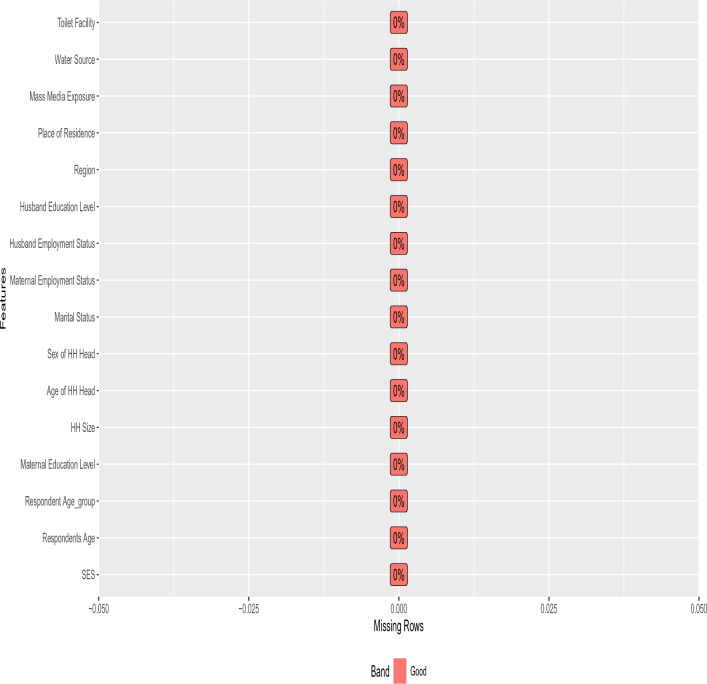
Variables of the dataset used to develop the prediction models after cleaning.

Data cleaning is a crucial step in ensuring the quality and reliability of the dataset. In this study, a thorough data cleaning process was conducted to identify and rectify errors, inconsistencies, and outliers. Duplicate entries were removed, formatting issues were corrected, and data entry errors were addressed. The data cleaning process resulted in a cleaned dataset that formed the basis for subsequent analyses, as shown in Figure 1.

#### Missing values imputation

Missing Value imputation is a critical step in data preprocessing to address the issue of missing data. This imputation process was carried out iteratively until a 100% completeness of all variables was achieved. This rigorous approach aimed to minimize the impact of missing data on subsequent analyses and ensure the reliability of the results. Thus, the dataset became more robust, allowing for more accurate analysis and interpretation.

In summary, missing value imputation was performed using appropriate techniques to handle missing data, resulting in a more complete dataset suitable for further analysis, as shown again in Figure 1.

### Feature selection

Feature selection techniques were then employed to identify the most relevant predictors of poverty. This involved analyzing the correlation between variables, conducting exploratory data analysis, and applying statistical tests to determine the significance and predictive power of each variable.

To identify the most relevant predictors of poverty, we employed a comprehensive set of feature selection techniques. Our methodology involved several steps to ensure a thorough analysis of the data.

First, we conducted a correlation plot to examine the relationships between variables. This helped us identify potential associations and dependencies among the predictors. Additionally, we performed descriptive analysis to gain insights into the distribution and summary statistics of the variables.

Next, we applied inferential analysis using classical regression models, specifically logistic regression. In this way, we assessed the significance and predictive capability of each variable in relation to poverty. This allowed us to understand the individual contributions of the predictors and identify statistically significant relationships.

In addition to classical regression models, we implemented four ML algorithms: RF, DT, SVM, and logistic regression. These algorithms provided a more robust and comprehensive analysis of the data, capturing complex relationships and non-linear interactions.

After evaluating the performance of the different ML algorithms, we selected RF as our final approach for feature selection. We recall that RF is an ensemble learning method that combines multiple decision trees to estimate feature importance. By leveraging the collective predictive power of these trees, RF assigns importance scores to each variable, enabling us to rank and select the most influential predictors of poverty.

By incorporating correlation analysis, descriptive analysis, inferential analysis using classical regression models, and four ML algorithms, we employed a rigorous and multi-faceted approach to feature selection. This comprehensive methodology ensured that we identified the most relevant predictors of poverty, enhancing the accuracy and interpretability of our subsequent analyses.

### Machine learning methods

To predict poverty in Somalia, several ML algorithms were employed, including the RF, DT, SVM, and logistic regression. These algorithms were implemented using the R software version 4.1.2. The ML models were trained on the preprocessed dataset, with poverty status as the target variable and a set of selected predictor variables.

For the RF algorithm, we utilized the randomForest package in R. We conducted parameter tuning by adjusting the number of trees, maximum depth, minimum node size, and other relevant parameters to optimize the RF model’s performance.

Similarly, for the DT algorithm, we utilized the rpart package in R. We conducted parameter tuning by varying the maximum depth, minimum split, and other relevant parameters to identify the optimal settings for the DT model.

For the SVM algorithm, we employed the e1071 package in R. We conducted a grid search combined with cross-validation to determine the optimal values for the hyperparameters, such as the kernel type, cost, and gamma.

Regarding the logistic regression, we utilized the glm function in R. Parameter tuning for logistic regression involved adjusting the regularization parameter and other relevant parameters to optimize the model’s performance.

### Model evaluation

The performance of the predictive models was assessed using various evaluation metrics. These included the confusion matrix, accuracy, precision, sensitivity, specificity, recall, F1 score, and the AUROC curve. The confusion matrix provided a comprehensive overview of the model’s predictive performance, while accuracy measured the overall correctness of the predictions. Precision, sensitivity, and specificity provided insights into the model’s ability to correctly identify positive and negative instances. The AUROC curve indicated the model’s discrimination power between positive and negative instances.

In addition, cross-validation is a widely used method for assessing the generalizability of ML models. It helps mitigate issues such as overfitting by providing a more robust estimation of the model’s performance on unseen data. Specifically, we utilized *k*-fold cross-validation, where the dataset is divided into *k* subsets, or folds. The models were trained on $(k-1)$ folds and evaluated on the remaining fold. This process was repeated *k* times, rotating the evaluation fold each time. The performance metrics reported in our evaluation reflect the average performance across all folds, ensuring a more reliable and unbiased estimation of the models’ predictive capabilities^4^.

### Ethical considerations

Acquiring participants for this study is impossible since all personally identifiable information has been removed from the dataset. However, permission to utilize the data was obtained from the Somalia National Bureau of Statistics. Hence, obtaining additional ethical approval may not be necessary.

### Statistical analysis

In addition to the ML methods, conventional statistical analysis was performed using STATA version 17. Descriptive statistics were computed to summarize the characteristics of the sample population. A regression analysis was conducted to examine the relationship between predictor variables and poverty outcomes, utilizing appropriate statistical tests and controlling for potential confounding factors.

Logistic regression analysis was employed to model the binary nature of the outcome variable. It is a widely used statistical technique specifically designed for binary outcomes. By utilizing logistic regression, we can effectively analyze the relationship between the independent variables and the binary outcome variable.

To ensure the validity of our logistic regression models, we considered the assumptions specific to this analysis. We assessed the assumption of linearity in the logit by examining the relationship between the log odds of the outcome and the independent variables. Additionally, we verified the assumption of independence of observations, which assumes that the observations are not correlated with each other.

In order to account for potential confounding factors, we carefully selected relevant variables based on prior knowledge and existing literature. These confounding factors were included as independent variables in the logistic regression models to control for their influence on the outcome variable. By considering these factors, we aim to accurately estimate the relationship between the independent variables and the binary outcome while mitigating the impact of potential confounding effects.

### Ethics approval and consent to participate

The research utilized secondary data obtained in accordance with the National Data Sharing and Accessibility Policy (NDSAP) implemented by the Government of Somalia. The dataset employed in the study did not contain any personally identifiable information about the survey participants, thus eliminating the need for ethical approval for this study.

## Results

### Descriptive statistics

The analyzed dataset allows us to gain insights into various socio-economic aspects of the surveyed population in Somalia.

#### Descriptive statistics for the categorical predictor variables

We examined several categorical explanatory variables to better understand the determinants of poverty in the country, as shown in Table 1.

Among the regions, the highest number of respondents (3111; 9.632%) were from Banadir, followed by Sanag (2893; 8.957%). These regional distributions will help us understand the geographical representation of our sample and its implications for poverty rates.

Residence is another important factor. We observed that a significant proportion of respondents lived in urban areas (12,410; 38.5%), while a considerable number resided in nomadic settings (11,117; 34.42%). This will enable us to explore the differences in poverty prevalence between urban and nomadic populations and their potential impact on poverty-related variables.

Access to basic amenities is crucial in understanding poverty dynamics. The majority of respondents had improved water sources (19,039; 58.947%) and improved toilet facilities (12,554; 38.869%), while a notable proportion relied on unimproved sources (41.05%) and unimproved facilities (61.13%). These findings will contribute to our analysis of the relationship between access to basic services and poverty levels in Somalia.

Educational attainment plays a significant role in poverty reduction. The majority of mothers had no formal education (28,120; 87.06%), while a smaller percentage completed primary education (3215; 9.954%). In our study, we will examine the influence of maternal education on poverty outcomes.

Gender dynamics are also important to consider. The household head was predominantly male (21,431; 66.35%), while female heads accounted for 10,867 (33.64%). We will explore the potential role of gender in poverty determination and its interaction with other variables.

Moreover, employment patterns among mothers and husbands were examined. Only a small proportion of mothers were employed (366; 1.133%), while the majority were unemployed (31,932; 98.866%). Among husbands, 43.299% were employed (13,985), while 56.70% were unemployed (18,313). These employment figures will help us understand the relationship between household income and poverty status.

By considering these categorical explanatory variables from the SDHS 2020 dataset, our study aims to employ ML algorithms to identify the key determinants of poverty in Somalia. We will compare the predictive performance of new contenders with classical models, aiming to provide valuable insights into poverty dynamics and contribute to poverty alleviation efforts in the country.

**Table 1:** Frequency distribution of categorical variables.

Variable	Frequency	Percentage (%)
Wealth index		
Relatively well	15,112	46.79
Relatively poor	17,186	53.21
Region		
Awdal	1577	4.9
Waqooyigalbed	2410	7.461
Togdheer	2565	7.941
Sool	2973	9.20
Sanag	2893	8.957
Bari	2013	6.232
Nugal	1822	5.641
Mudug	1776	5.498
Galdaduud	1675	5.186
Hiran	1448	4.483
Middleshabelle	1661	5.142
Banadir	3111	9.632
Bay	579	1.792
Bakol	2054	6.359
Gedo	1919	5.941
Lower juba	1822	5.641
Place of residence		
Rural	8771	27.2
Urban	12,410	38.5
Nomadic	11,117	34.42
Water source		
Improved	19,039	58.947
Unimproved	13,259	41.05
Toilet facility		
Improved	12,554	38.869
Unimproved	19,744	61.13
Maternal education		
No education	28,120	87.06
Primary	3215	9.954
Secondary	796	2.464
Higher	167	0.517
Sex of household head		
Male	21,431	66.35
Female	10,867	33.64
Maternal employment		
Employment	366	1.133
Unemployment	31,932	98.866
Husband employment		
Employment	13,985	43.299
Unemployment	18,313	56.70
Husband Education		
No education	25,763	79.766
Primary	2480	7.678
Secondary	2530	7.833
Higher	1525	4.7216

#### Descriptive statistics of the continuous predictor variables

The descriptive statistics for the continuous variables are presented in Table 2. The minimum number of household members was 0, while the maximum was 9. The average household size was 5.3, with a standard deviation of 2.17. The age of the household head ranged from 15 years to 49 years, with a mean of 38.178 years and a standard deviation of 21.80.

**Table 2:** Descriptive statistics: continuous predictor variables.

Variable	Min	1st Quartile	Median	Mean	Variance	Skewness	3rd Quartile	Kurtosis	Max
Household size	0	4	5	5.3	4.71	-0.07137	7.0	2.156	9
Age of household head	15	22	23	38.178	475.48	0.26509	41	1.58	49

### Correlation analysis

**Figure 2:**
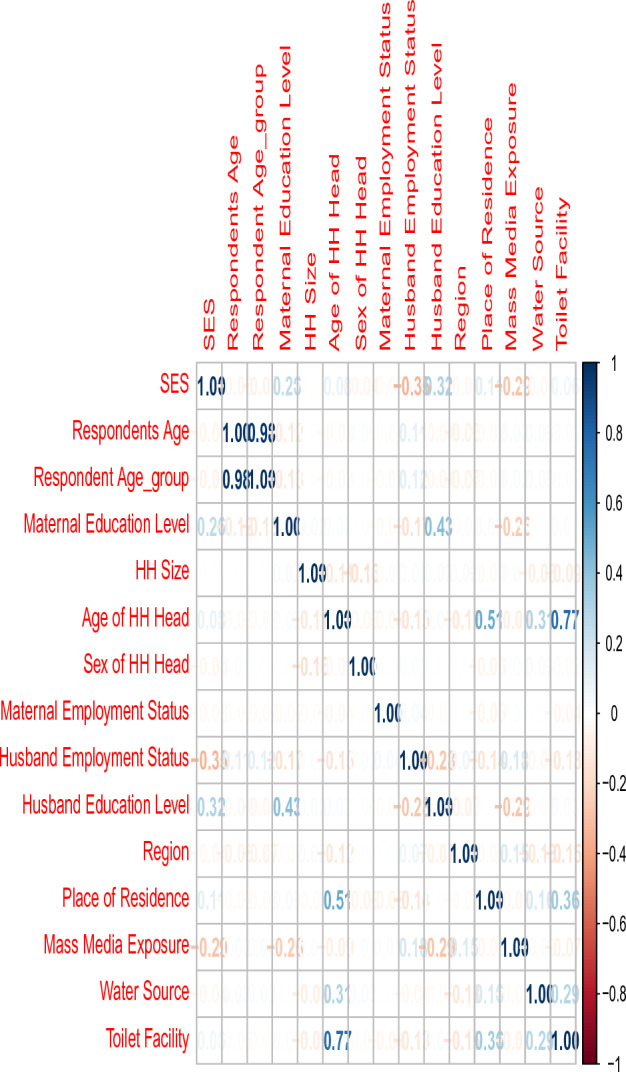
Correlation plot for the variables of the study.

A correlation analysis was conducted to examine the relationships between variables in our feature selection process. The correlation coefficients were computed and visualized in a correlation plot, which provided a comprehensive overview of the pairwise correlations. By analyzing the plot, we identified variables with strong positive or negative correlations and considered potential multicollinearity issues. This analysis served as a valuable initial step, guiding our subsequent feature selection by highlighting the most relevant predictors of poverty. The findings from the correlation analysis were summarized in the correlation plot shown in Figure 2, enabling us to make informed decisions and enhance the overall effectiveness of our feature selection methodology.

### Inferential analaysis

The logistic regression analysis aimed to identify key factors associated with poverty in Somalia using the first-ever SDHS dataset from 2020. Several variables were examined, including age group, maternal education, household size, age of the household head, sex of the household head, maternal employment status, husband employment status, husband education, region, place of residence, water source, and toilet facility.

The results revealed significant associations between certain variables and the odds of poverty, as presented in Table 3. Individuals with primary education had nearly three times higher odds of poverty (OR = 2.95, 95% CI [1.59, 5.50]), while those with secondary education exhibited significantly higher odds (OR = 26.60, 95% CI [11.24, 63.09]) compared to individuals with no education. Similarly, individuals with higher education had 8.49 times higher odds of poverty (OR = 8.49, 95% CI [1.57, 45.86]).

Female-headed households had 0.80 times lower odds of poverty (OR = 0.80, 95% CI [0.67, 0.95]) compared to male-headed households. However, household size (OR = 0.99, 95% CI [0.97, 1.01]), age of the household head (OR = 1.00, 95% CI [0.98, 1.01]), and maternal employment status (OR = 0.99, 95% CI [0.80, 1.23]) did not show statistically significant associations with poverty.

Regarding husband-related variables, households with unemployed husbands demonstrated substantially higher odds of poverty (OR = 3.07, 95% CI [1.78, 5.31]) compared to households with employed husbands. However, husbands’ education did not exhibit significant associations with poverty.

Geographically, the analysis revealed variations in the odds of poverty across different regions of Somalia. Each region had its own odds ratio, indicating the likelihood of poverty in that specific region compared to the reference region (Awdal). However, the interpretation of these regional odds ratios requires further context and examination.

In terms of place of residence, individuals living in urban areas had slightly lower odds of poverty (OR = 0.89, 95% CI [0.77, 1.03]) compared to those in rural areas, while individuals residing in nomadic areas had 1.60 times higher odds of poverty (OR = 1.60, 95% CI [1.11, 2.32]).

Furthermore, households with unimproved water sources (OR = 1.51, 95% CI [1.20, 1.91]) and those with unimproved toilet facilities (OR = 1.24, 95% CI [1.04, 1.48]) exhibited increased odds of poverty compared to households with improved water sources and toilet facilities, respectively.

In summary, these findings highlight the multifaceted nature of poverty in Somalia and underscore the importance of addressing factors such as education, gender dynamics, and access to basic amenities in efforts to alleviate poverty levels in the country.

**Table 3:** Logistic regression model analysis of key determinants associated with poverty.

Variable	Odds	SE	Z-value	*P*-value	Lower 95% CI	Upper 95% CI
Age group						
15–19	Reference					
20–24	1.087327	0.1217815	0.75	0.455	0.8730216	1.35424
25–29	1.279387	0.1372602	2.30	**0.022**	1.036764	1.036764
30–34	1.38871	0.1491225	3.06	**0.002**	1.125144	1.714018
35–39	1.143936	0.1225597	1.26	0.209	0.9272675	1.411231
40–44	1.018682	0.1117848	0.17	0.866	0.8215464	1.263121
45–49	1.28251	0.1479415	2.16	**0.031**	1.022992	1.607865
Maternal education						
No education	Reference					
Primary	2.998338	0.1704348	19.32	**0.000**	2.682228	3.351703
Secondary	26.59666	8.386415	10.40	**0.000**	14.33599	49.34312
Higher	8.485417	5.130254	3.54	**0.000**	2.594416	27.7528
Household size	0.9850939	0.0061927	-2.39	**0.017**	0.9730309	0.9973065
Age of household head	1.002767	0.0011735	2.36	**0.018**	1.00047	1.00507
Sex of household head						
Female	Reference					
Male	.8018376	0.0231724	-7.64	**0.000**	0.7576829	0.8485655
Maternal employment status						
Yes	Reference					
No	0.9981961	0.1282396	-0.01	0.989	.7759984	1.284017
Husband employment status						
Yes	Reference					
No	0.3218246	0.0091556	-39.85	**0.000**	0.3043711	0.3402789
Husband education						
No ed (Ref)						
Primary	1.439087	0.0761774	6.88	**0.000**	1.297266	1.596412
Secondary	5.105135	0.3678985	22.62	**0.000**	4.432675	5.879611
Higher	12.18714	1.886552	16.15	**0.000**	8.997817	16.50695
Region						
Awdal	Reference					
Waqooyigalbed	1.13132	0.0894105	1.56	0.118	0.968977	1.320863
Togdheer	3.525278	0.2778832	15.98	**0.000**	3.020624	4.114244
Sool	2.953598	0.2288887	13.98	**0.000**	2.537392	3.438073
Sanag	2.514173	0.1950631	11.88	**0.000**	2.159506	2.92709
Bari	1.047298	0.0843198	0.57	0.566	0.894	1.226315
Nugal	1.140189	0.0949141	1.58	0.115	0.9685438	1.342253
Mudug	1.006054	0.0835948	0.07	0.942	0.8548567	1.183993
Galdaduud	1.345585	0.1104544	3.62	**0.000**	1.145616	1.580459
Hiran	1.065255	0.091278	0.74	0.461	0.9005693	1.260057
Middle shabelle	1.49944	0.1242415	4.89	**0.000**	1.274676	1.763837
Banadir	3.092402	0.2402945	14.53	**0.000**	2.655544	3.601127
Bay	8.065486	0.9646898	17.45	**0.000**	6.380001	6.380001
Bakol	1.955825	0.1556497	8.43	**0.000**	1.673359	2.285972
Gedo	.6609969	0.055718	-4.91	**0.000**	0.5603358	.7797413
Lower juba	2.959751	0.2430127	13.22	**0.000**	2.519803	3.476513
Place of residence						
Rural (ref)						
Urban	.8867439	0.034032	-3.13	**0.002**	0.8224893	0.9560182
Nomadic	1.596815	0.0696994	10.72	**0.000**	1.465887	1.739437
Water source						
Improved	Reference					
Unimproved	.6567353	0.020311	-13.60	**0.000**	0.618109	0.6977753
Toilet facility						
Improved	Reference					
Unimproved	.8566099	0.0374347	-3.54	**0.000**	0.7862936	0.9332143
Intercept	16.6144	3.748576	12.46	**0.000**	10.67664	25.8544

Significant values are in [bold].

### Machine learning models performance and predicting poverty

Table 4 provides various evaluation metrics to assess the performance of each predictive model in predicting poverty. These metrics include accuracy, recall, sensitivity, specificity, positive predictive value, negative predictive value, precision, F1 score, prevalence, detection rate, detection prevalence, and balanced accuracy.

Among the models, RF achieved the highest accuracy at 96.38%, followed by the logistic regression at 74.95%, DT at 73.73%, and SVM at 67.21%. The RF also demonstrated the highest recall (95.90%) and sensitivity (95.90%), indicating its ability to correctly identify the majority of individuals experiencing poverty. The logistic regression had a recall of 70.49%, while the DT and SVM had lower recall values of 66.44% and 62.49%, respectively.

In terms of specificity, the RF performed the best at 96.80%, followed by the DT at 86.17%, logistic regression at 80.09%, and SVM at 73.45%. These values indicate the models’ ability to correctly identify non-poor individuals.

The positive predictive value (also known as precision) measures the proportion of correctly predicted poor individuals among all predicted poor cases. The RF achieved the highest positive predictive value at 96.41%, followed by the logistic regression at 80.34%, SVM at 75.66%, and DT at 89.13%. The negative predictive value measures the proportion of correctly predicted non-poor individuals among all predicted non-poor cases. The RF had the highest negative predictive value at 96.35%, followed by the logistic regression at 70.17%, DT at 60.06%, and SVM at 59.71%.

The F1 score, which balances precision and recall, was highest for the RF at 96.16%, followed by the DT at 76.13%, logistic regression at 75.09%, and SVM at 68.44%. These scores indicate the overall performance of the models in capturing both the positive and negative classes. The prevalence indicates the proportion of individuals experiencing poverty in the dataset. The logistic regression had a prevalence of 53.57%, followed by the DT at 63.06%, SVM at 56.91%, and RF at 47.25%.

The detection rate measures the proportion of correctly predicted poor individuals among all actual poor cases. The RF achieved the highest detection rate at 45.32%, followed by the DT at 41.90%, logistic regression at 37.76%, and SVM at 35.56%. The detection prevalence represents the proportion of predicted poor individuals among all individuals in the dataset. All models had a detection prevalence of 47.00%.

**Figure 3:**
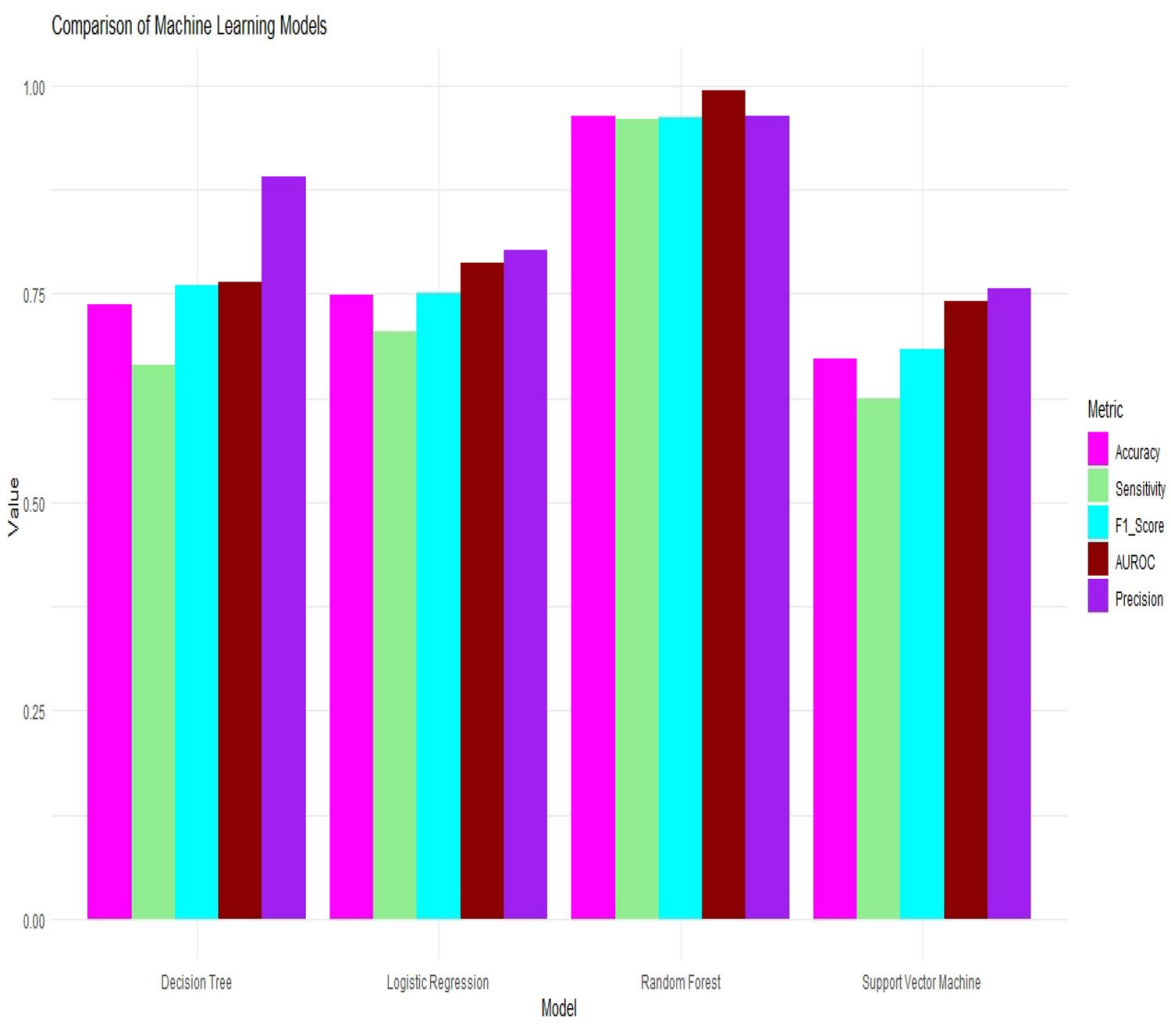
Model comparison metrics for logistic regression, DT, RF and SVM: accuracy, F1 score, precision, AUROC, and sensitivity.

Finally, balanced accuracy provides an average of sensitivity and specificity, giving equal weight to both classes. The RF had the highest balanced accuracy at 96.35%, followed by the DT at 76.30%, logistic regression at 75.29%, and SVM at 67.97%. In summary, the RF outperformed the other models in terms of accuracy, recall, sensitivity, specificity, positive predictive value, negative predictive value, precision, F1 score, and balanced accuracy. However, it’s important to consider other factors, such as model complexity, interpretability, and computational requirements, when choosing the most appropriate predictive model for a specific context. Additionally, in Figure 3, we present a comprehensive comparison of model performance metrics for ML models. The metrics evaluated include accuracy, F1 score, precision, area under the receiver operating characteristic curve (AUROC), and sensitivity. This figure provides a visual summary of the performance of each algorithm, allowing for a quick and insightful comparison of their predictive capabilities.

**Table 4:** Predictive models’ performance.

Evaluation matrix			Predictive models							
			Logistic regression		RF		DT		SVM	
Confusion matrix			Predicted		Predicted		Predicted		Predicted	
			Poor	Well	Poor	Well	Poor	Well	Poor	Well
Observed	Poor		3442	2114	4283	1273	4184	1372	3454	2102
	Well		2215	3472	493	5194	536	5151	1425	4262
			%	%			%	%		
Accuracy			74.95	96.38			73.73	67.21		
Recall			70.49	95.90			66.44	62.49		
Sensitivity			70.49	95.90			66.44	62.49		
Specificity			80.09	96.80			86.17	73.45		
Positive predictive value			80.34	96.41			89.13	75.66		
Negative predictive value			70.17	96.35			60.06	59.71		
Precision			80.34	96.41			89.13	75.66		
F1 score			75.09	96.16			76.13	68.44		
Prevalence			53.57	47.25			63.06	56.91		
Detection rate			37.76	45.32			41.90	35.56		
Detection prevalence			47.00	47.00			47.00	47.00		
Balanced accuracy			75.29	96.35			76.30	67.97		

**Figure 4:**
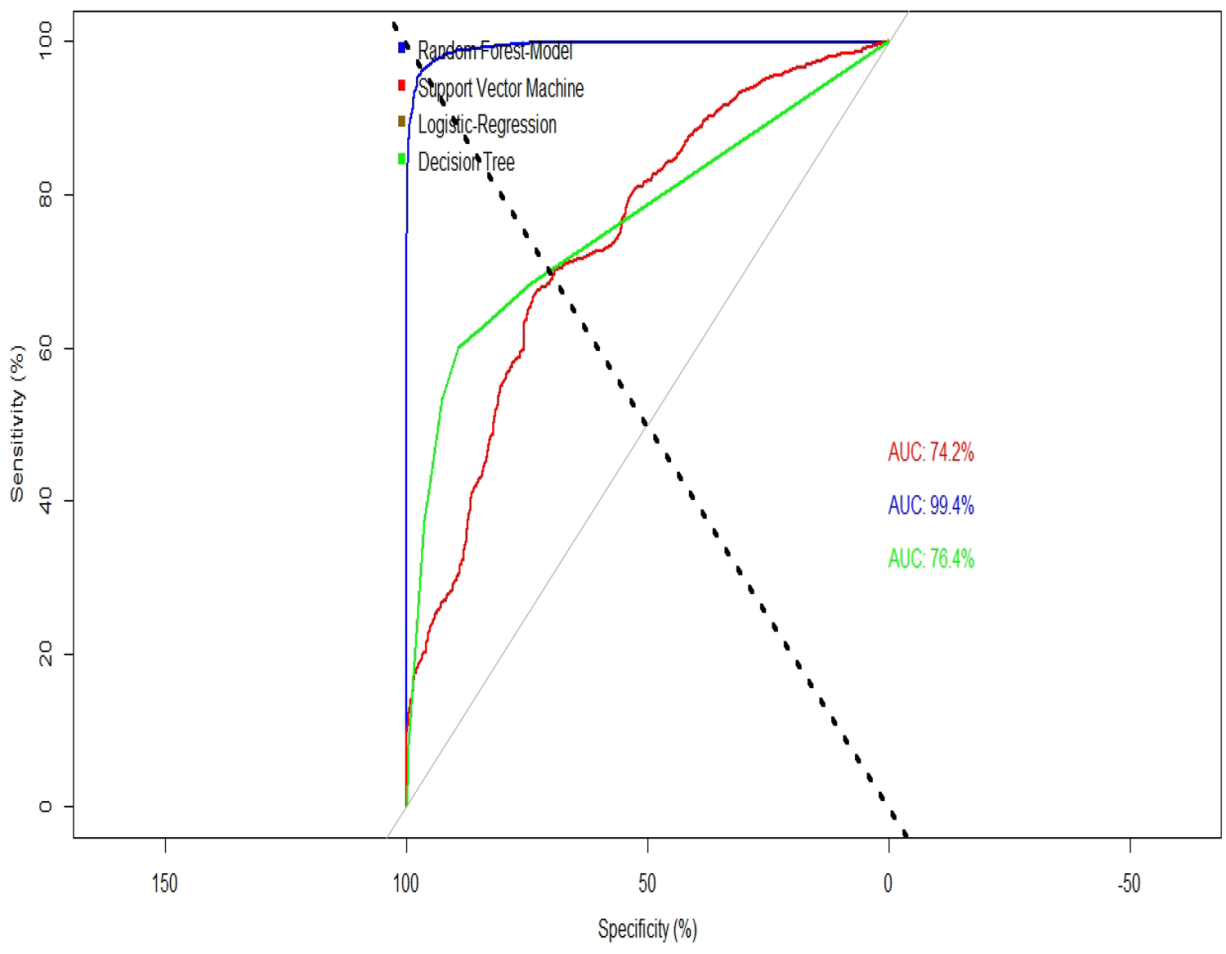
AUROC curves for the four competitive ML models.

Figure 4 illustrates the AUROC curve visualization in this study. Among the four ML models utilized, the ROC curve of the RF model exhibits the highest area under the curve (AUC) value. This signifies that the RF model outperforms the other models in accurately classifying cases as either poor or well-off.

### Importance features selection

**Figure 5:**
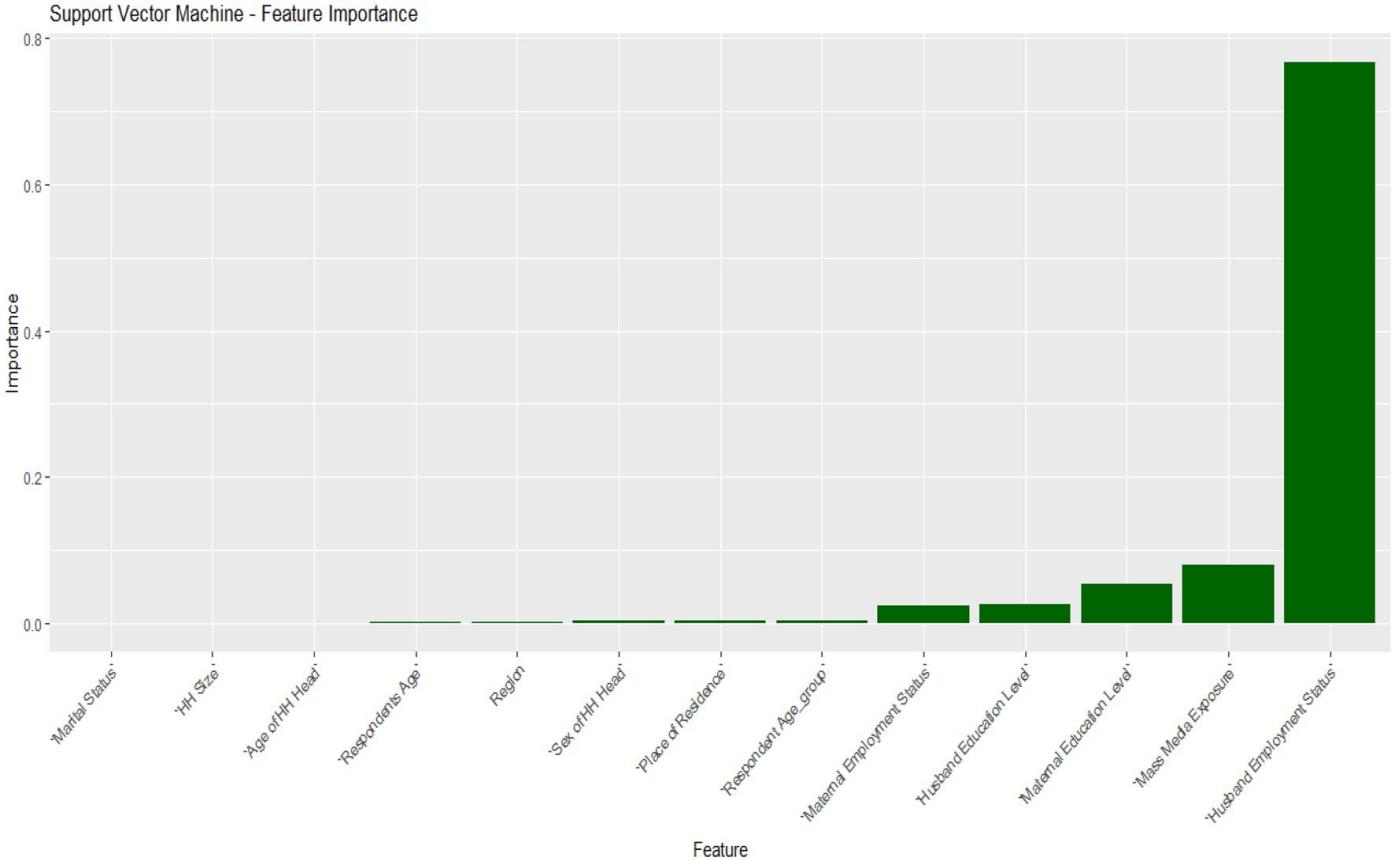
Important features selected for the SVM model.

**Figure 6:**
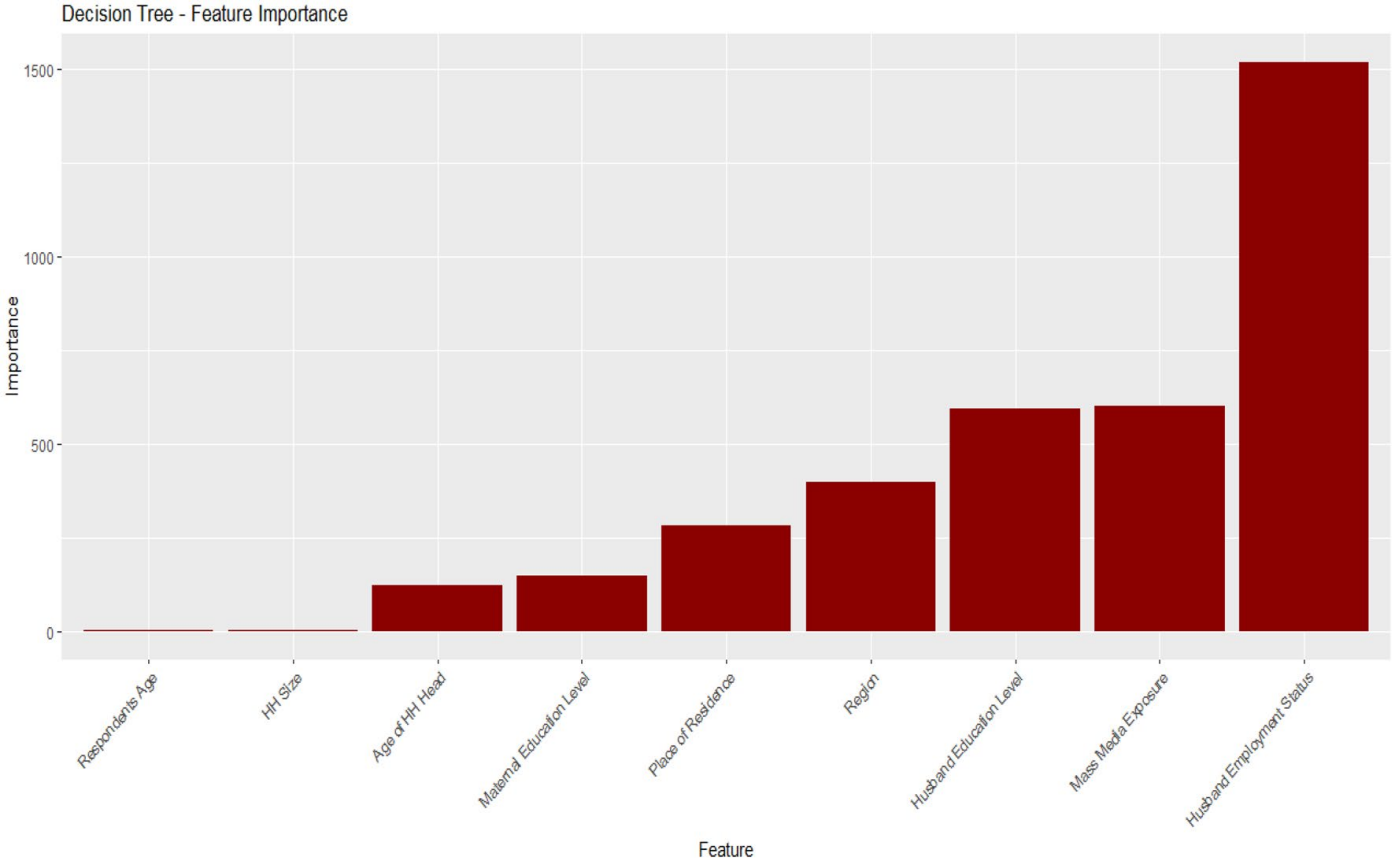
Important features selected for the DT model.

**Figure 7:**
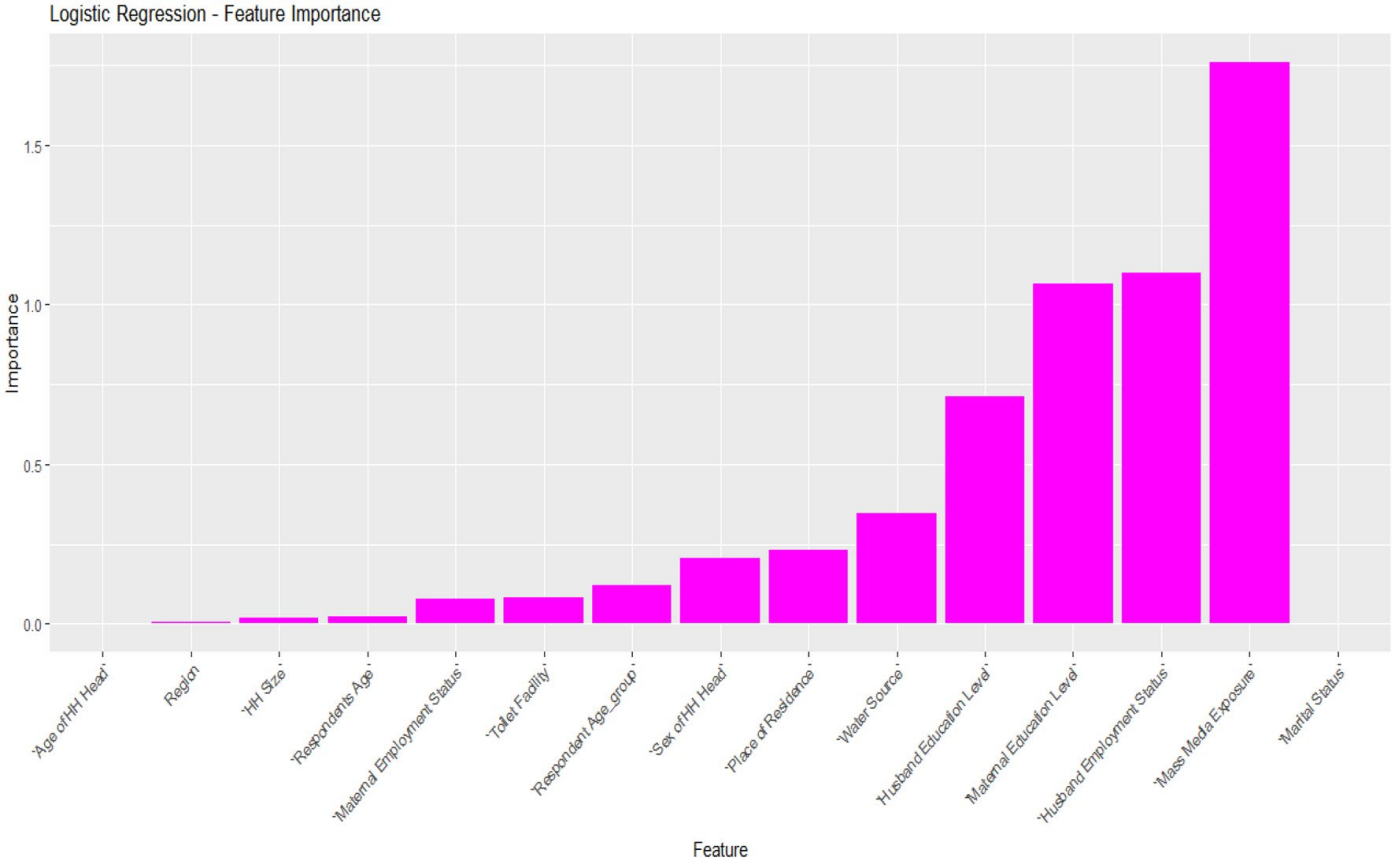
Important features selected for the logistic regression model.

**Figure 8:**
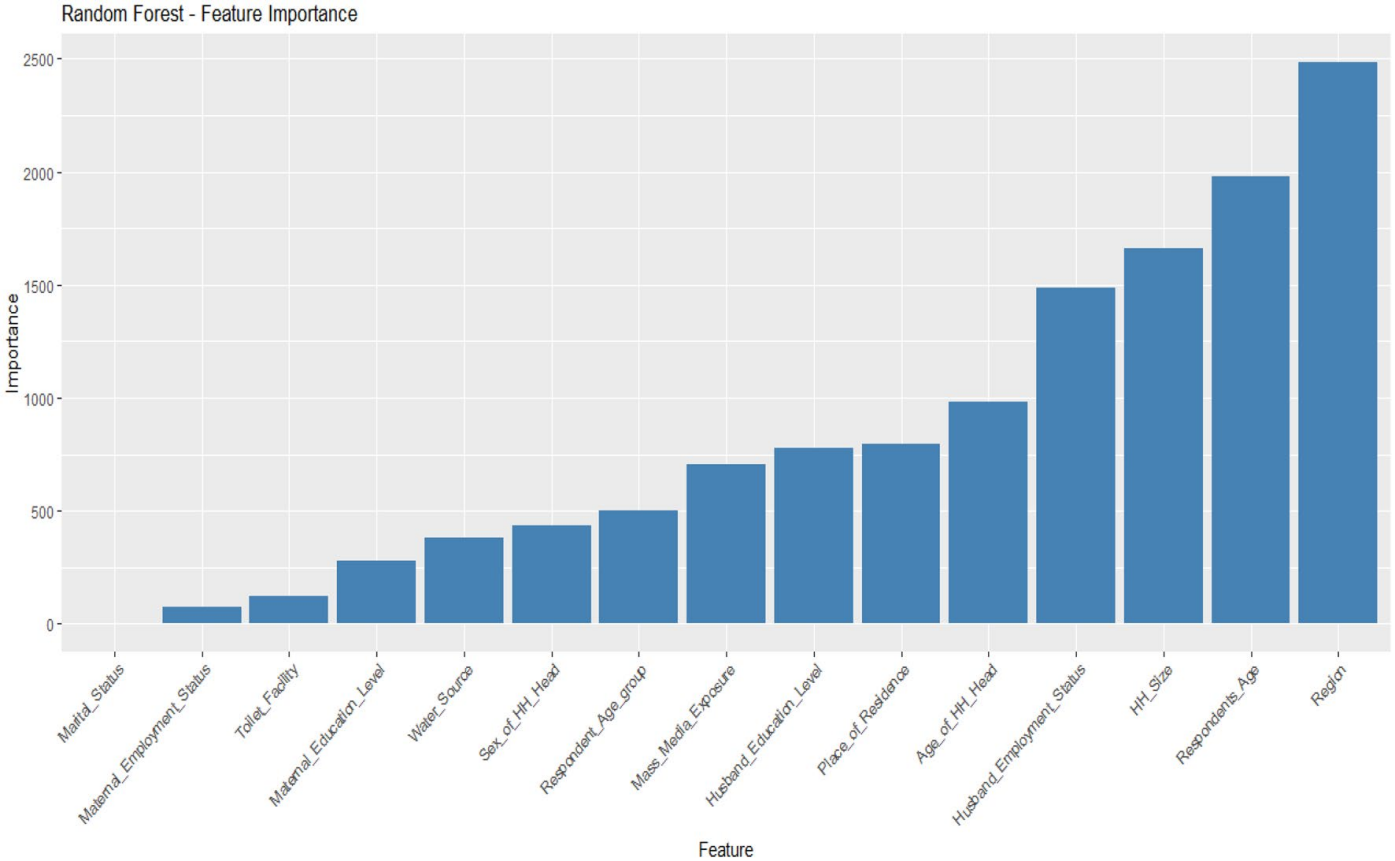
Important features selected for the RF model.

In this study, we investigated the feature selection process using four popular ML algorithms: RF, DT, SVM, and logistic regression. Each algorithm was utilized to assess the importance and relevance of features in the dataset. By comparing the results of these four models, we aimed to identify the most informative features for our analysis. The feature selection process plays a crucial role in enhancing the performance and interpretability of ML models. Through this investigation, we aimed to gain insights into the relative strengths and limitations of each algorithm in terms of feature selection. This knowledge will contribute to making informed decisions regarding the inclusion or exclusion of features in subsequent analyses and modeling tasks. All the feature selections are summarized in Figs. 5, 6, 7, and 8.

After evaluating the feature selection results obtained from the RF, DT, SVM, and logistic regression, we chose to prioritize the RF for several reasons. The RF demonstrated superior performance across multiple evaluation metrics, including accuracy, sensitivity, AUROC, F1 score, precision, and other relevant metrics. Its ability to handle high-dimensional data, capture complex interactions, and be robust to outliers made it a compelling choice. Additionally, RF’s built-in feature importance calculation based on metrics like Gini impurity or mean decrease in accuracy provided valuable insights into the relevance and significance of features. The overall combination of its excellent performance and comprehensive feature importance analysis solidified our decision to consider RF as the primary feature selection method for our study.

Thus, in our statistical context, we employed a RF classifier (Fig. 5) to identify significant features associated with poverty. The analysis revealed a set of 13 key features that contribute to poverty, including administrative region, household size, age group of respondents, employment status of the husband, age of the household head, place of residence, education level of the husband, exposure to mass media, sex of the household head, source of drinking water, maternal education, type of toilet facility, and employment status of the mother. These findings highlight the complex nature of poverty and underscore the importance of considering various socio-economic factors when addressing poverty-related issues. The insights gained from this feature selection process, combined with RF’s excellent performance and comprehensive feature importance analysis, provide a solid foundation for further analysis and the development of targeted interventions to alleviate poverty in the studied population.

## Discussion

The logistic regression analysis aimed to identify key factors associated with poverty in Somalia using the first-ever SDHS dataset from 2020. Our results revealed significant associations between certain variables and the odds of poverty. Individuals with primary education had nearly three times higher odds of poverty, while those with secondary education exhibited significantly higher odds. Female-headed households had lower odds of poverty compared to male-headed households. Unemployed husbands and households with unimproved water sources or toilet facilities demonstrated increased odds of poverty. Geographical variations in the odds of poverty were observed across different regions of Somalia. These findings emphasize the multifaceted nature of poverty in Somalia and highlight the importance of addressing education, gender dynamics, and access to basic amenities in poverty alleviation efforts.

In comparing our study findings to previous research, it is important to note that limited studies have specifically focused on predicting poverty in Somalia using advanced ML techniques. However, our results align with previous studies conducted in similar contexts.

Regarding the association between education and poverty, our results are consistent with prior studies that have identified education as a significant determinant of poverty in developing countries^36^. Individuals with higher levels of education generally have better employment prospects and income-earning opportunities, reducing their likelihood of experiencing poverty.

The finding that female-headed households exhibit lower odds of poverty aligns with existing literature on gender and poverty^37,38^. Female-headed households often face additional challenges, such as limited access to resources and economic opportunities. However, our results suggest that these households may have developed strategies for resilience and economic empowerment, leading to lower poverty rates compared to male-headed households.

The association between household characteristics (such as household size and age of the household head) and poverty found in our study is consistent with previous research that highlights the complex interplay between household dynamics and poverty^23,39^. While we did not find statistically significant associations for these variables, their potential influence on poverty cannot be overlooked, and further research could explore their nuanced effects.

The regional disparities in poverty rates identified in our study align earlier research that has documented spatial variations in poverty within countries^40,41^. This suggests the need for targeted regional policies and interventions to address localized poverty challenges and promote equitable development.

The association between access to basic amenities (water sources and toilet facilities) and poverty is consistent with studies emphasizing the importance of infrastructure and sanitation in poverty reduction^37^. Lack of access to improved water and sanitation facilities can exacerbate health and economic vulnerabilities, contributing to higher poverty rates.

While our study contributes to the understanding of poverty determinants in Somalia, further research is needed to expand upon these findings and compare them with a broader range of studies examining poverty dynamics in similar contexts.

We also aimed to apply ML algorithms to identify the key determinants of poverty in Somalia using the first-ever SDHS 2020 dataset. The performance of various predictive models, including logistic regression, RF, DT, and SVM, was evaluated and compared. The findings provide insights into the effectiveness of these models in predicting poverty and offer implications for poverty alleviation strategies in Somalia.

The RF model emerged as the top-performing model in this analysis. It achieved the highest accuracy (96.38%), indicating its ability to make correct predictions for a significant portion of the dataset. The model also demonstrated high recall (95.90%), specificity (96.80%), precision (96.41%), and F1 score (96.16%), indicating its strong performance in identifying both the “Poor” and “Well” classes. These results suggest that the RF model is well-suited for identifying poverty determinants in Somalia and can potentially contribute to targeted interventions and poverty reduction efforts.

The logistic regression model also showed promise, although its performance was slightly lower than that of the RF model. With an accuracy of 74.95% and a recall of 70.49%, the logistic regression model displayed a reasonably good capacity for classifying instances. However, its specificity (80.09%) and precision (80.34%) were relatively lower, indicating a higher number of false positives. Nevertheless, the logistic regression model can still provide valuable insights into poverty determinants and contribute to an understanding of the factors driving poverty in Somalia.

The DT model exhibited competitive performance, with an accuracy of 73.73% and a recall of 66.44%. It demonstrated relatively high specificity (86.17%) and precision (89.13%), showcasing its ability to effectively identify the “Well” class. However, the model had a lower recall and F1 score compared to the RF model, implying a higher number of false negatives. Despite this limitation, the DT model can still offer valuable insights into the key determinants of poverty in Somalia.

In contrast, the SVM model demonstrated the lowest overall performance among the evaluated models. With an accuracy of 67.21% and a recall of 62.49%, the SVM model struggled to accurately classify instances. Its specificity (73.45%), precision (75.66%), and F1 score (68.44%) were also relatively lower compared to the other models. While the SVM model may have limitations in predicting poverty determinants in Somalia, it can still contribute to the overall understanding of the problem and provide additional perspectives.

It is important to consider the prevalence of positive instances in the dataset when interpreting the results. The DT model had the highest prevalence value (63.06%), indicating a higher proportion of instances belonging to the “Well” class. On the other hand, the RF model had the lowest prevalence value (47.25%), suggesting a more balanced distribution of the two classes. These prevalence values have implications for the generalizability of the findings and should be taken into account when designing targeted poverty reduction interventions.

Overall, the results of this study highlight the potential of ML algorithms, particularly the RF model, in identifying the key determinants of poverty in Somalia. The findings can inform policymakers and stakeholders involved in poverty alleviation efforts, providing them with valuable insights into the factors driving poverty and enabling them to develop more effective strategies. However, it is crucial to acknowledge the limitations of the study, such as the reliance on a single dataset and the need for further research to validate and expand upon these findings. Future studies can explore additional variables, consider alternative models, and incorporate external data sources to enhance the accuracy and robustness of poverty prediction models in Somalia.

In addition, future research could consider incorporating various other factors to enhance the understanding of poverty determinants in Somalia. These may include variables such as household assets, access to electricity and clean energy, food security and nutrition status, geographical location and proximity to essential services, gender and household composition, quality of housing and infrastructure, social and cultural factors influencing poverty, access to financial services and credit opportunities, exposure to conflict and violence, as well as government policies and interventions related to poverty alleviation. By examining these variables in future studies, we gain a more comprehensive perspective on the complex dynamics of poverty in Somalia. This deeper understanding can contribute to the development of targeted interventions and policies aimed at addressing poverty challenges effectively and improving the overall well-being of the Somali population.

## Conclusion

In conclusion, this comprehensive study utilized logistic regression analysis to identify the significant determinants of poverty in Somalia. The findings highlight the crucial roles played by various factors, including age group, maternal education, household size, age and sex of the household head, maternal and husband employment status, husband education, region, place of residence, water source, and toilet facility, in shaping poverty outcomes. These insights offer valuable guidance to policymakers and stakeholders in designing targeted interventions and policies aimed at reducing poverty and fostering inclusive socioeconomic development in the Somali context.

Moreover, the study conducted a rigorous performance evaluation of different predictive models, encompassing logistic regression, RF, DT, and SVM. By utilizing the provided confusion matrix, the results indicate that the RF model exhibited the highest accuracy (96.38%) and specificity (96.80%) among the evaluated models, surpassing others in accurately predicting both poor and well-off outcomes. However, it is essential to consider interpretability and computational complexity when selecting the most suitable model for practical implementation.

To further enhance the understanding and application of these models, future research endeavors should focus on exploring the causal relationships between the identified determinants of poverty and poverty outcomes in Somalia. Additionally, efforts should be directed towards refining the predictive capabilities and overall performance of the models to effectively address the specific needs and complexities of the Somali context within the field of management science.

## Limitations of the study

It is important to acknowledge several limitations when interpreting the findings of this study. Firstly, the dataset used in the analysis is from 2020, collected before the COVID-19 pandemic. As a result, the data may not fully capture the current situation of households, particularly the impact of the pandemic on poverty. Future research should consider incorporating more recent data to validate and update the empirical results. Considering the potential implications of the pandemic strengthens the need for contextualizing and interpreting the study’s findings in light of the evolving poverty dynamics influenced by the crisis.

Secondly, while the RF was recommended by UN researchers for poverty prediction, it is important to note that different models may be more suitable for different situations. Therefore, the RF may not necessarily be the best model for poverty prediction in all contexts. Alternative models should be explored and compared to determine the most appropriate approach for poverty analysis.

Thirdly, while improving poverty prediction is crucial for poverty reduction and enhancing quality of life, identifying households at high risk of poverty is just one step in addressing the issue. The practical impact of poverty reduction relies on the implementation of targeted policies adopted to specific contexts, which may vary from one place to another. Therefore, the findings of this study should be considered in conjunction with the development and implementation of effective poverty reduction strategies.

Lastly, it is important to note that the findings of this study may not be generalizable to the entire Somali population. The study was conducted in sixteen states of the country, excluding Lower Shabelle and Middle Juba due to their specific characteristics. These regions were excluded because they were under AL-SHABAB (terrorists) administration during the data collection. Therefore, caution should be exercised when extrapolating the results to the entire population of Somalia.

Despite these limitations, this study is significant as it represents the first attempt to identify key determinants of poverty in Somalia using the first-ever SDHS data, which features a large sample size.

## Policy implications

The results of our study have important policy implications for addressing poverty in Somalia. Based on our findings, we propose the following recommendations:

1. Targeted Interventions: Given the high prevalence of poverty in Somalia, it is crucial to implement targeted interventions that specifically address the needs of vulnerable populations. Nomadic pastoralists, agro-pastoralists, and internally displaced persons (IDPs) were identified as groups with particularly high poverty rates. Therefore, policymakers should prioritize allocating resources and designing programs that cater to the unique challenges faced by these populations.
2. Regional Disparities: Geographical region was found to be a significant predictor of poverty. Policymakers should consider developing region-specific policies and strategies to reduce disparities. This may involve targeted investments in infrastructure, healthcare, education, and livelihood opportunities in regions with higher poverty rates. By addressing regional disparities, policymakers can ensure a more equitable distribution of resources and opportunities across the country.
3. Household Characteristics: Our analysis revealed that household size, age of the household head, and employment status of the husband are important predictors of poverty. To alleviate poverty, policymakers should focus on initiatives that improve access to family planning services, promote income-generating activities, and provide skill development opportunities. By addressing these household-level factors, policymakers can enhance the economic resilience of households and contribute to poverty reduction.
4. Urban Poverty: While urban areas in Somalia have a relatively lower poverty rate compared to rural areas, urban poverty is still a significant concern. Policymakers should implement targeted policies to address urban poverty, including initiatives that improve access to basic services, create employment opportunities, and ensure affordable housing options. These measures can help uplift urban communities and reduce poverty rates in urban areas.

By incorporating these policy implications into the decision-making process, policymakers and stakeholders can develop targeted and effective strategies for poverty reduction in Somalia. It is vital to consider these recommendations to ensure sustainable and inclusive development in the country.

## Data Availability

The dataset was accessed from https://microdata.nbs.gov.so/index.php/catalog/50. The data that support the findings of this study are available on request from the corresponding author.

## Abbreviations

AUC: Area under the curve
AUROC: Area under the receiver operating characteristic curve
DT: Decision tree
HH: Household
IDP: Internally displaced persons
ML: Machine learning
MMS: Mass media status
RF: Random forest
SDG: Sustainable development goals
SDHS: Somali demographic and health survey
SVM: Support vector machine
WI: Wealth index

## Code availability

The code or algorithm associated with this research will be made available to interested readers upon reasonable request from the corresponding author.
